# Assessing causes of death in the Cardiology Department of Yalgado Ouédraogo University Hospital

**DOI:** 10.11604/pamj.2014.19.155.5286

**Published:** 2014-10-15

**Authors:** Aristide Relwende Yameogo, Germain Mandi, Georges Millogo, Andre Samadoulougou, Patrice Zabsonre

**Affiliations:** 1 Service de Cardiologie du Centre Hospitalier Universitaire Yalgado Ouédraogo, Burkina Faso; 2 Unité de Formation et de Recherches en Sciences de la Santé (UFR/SDS), Université de Ouagadougou, Burkina Faso

**Keywords:** Causes of death, cardiovascular disease, immediate causes of death, Burkina Faso

## Abstract

**Introduction:**

Analysis of the underlying causes of death can develop action plans for prevention of death that could be avoided. The aim of our study was to analyse the causes of cardiovascular deaths in the cardiology department of Yalgado Ouedraogo University Hospital.

**Methods:**

The study was a descriptive retrospective study over a 24 month period among patients who died in the department.

**Results:**

Prevalence of death in the cardiology department was of 13.2%. Sex ratio was of 1.2 and 72.7% of patients were residing in Ouagadougou. Mean age of patients was 56.1 years and 59.4% of patients were under 65 years old. Hypertension was the major cardiovascular risk factor (46.1%) and 27.4% of patients had a medical history of dilated cardiomyopathy. Cardiogenic shock was the immediate cause of death in 55.5% of cases and the initial cause of death was hypertension and its complications in 46.1% of cases. Death was not notified in 18% of cases and no death had been medically certified.

**Conclusion:**

Death statistics are the most reliable data for public health interventions. However, it is necessary to establish an effective method of data gathering according to the WHO standards in order to facilitate international comparison.

## Introduction

Cardiovascular diseases are currently becoming a major health concern in developing countries due to their morbidity and mortality. This requires early interventions to counter this pattern [1]. Data on mortality represent one of the finest indicators that could guide and assess public health interventions [2]. However and in order to be useful, these data need to be reliable, complete, with an adequate data gathering system. In fact, many countries have a nice performance in deaths’ notifications according to the World Health Organization (WHO), but fail to have accurate information on death causes [3]. The “Local culture and maternal mortality” study conducted in Burkina Faso in 2008 found that data collected were not reliable. However, the study did not analyze the data gathering system [4]. The purpose of our study was to analyze the system of certification as well as medical causes of death in order to set up a more performant mechanism of notification and certification of death in the Cardiology department of Yalgado Ouedraogo University Hospital.

## Methods

We conducted a descriptive retrospective study over a 24 months period from January 1st 2011 to December 31^st^ 2012. All patients who died in the department during this study period were included. Data related to causes of death and their medical certification were gathered using both patients’ medical records and inpatient patients’ registry. We used the World Health Organization (WHO) International method of death certification to assess both patients’ records and inpatient registry. The morbidity process that lead to death was assessed using patients’ medical records. Death certification data that were not notified in patients’ records were considered as missing. Both underlying cause of death (the disease or injury which initiated the train of morbid events leading directly the death or the circumstances of the accident or violence which produced the fatal injury) and immediate causes of death (disease or condition directly leading the death) were defined according to the International Classification of diseases, 10th edition (ICD 10) [5]. Labelling and coding the initial causes of death were manually performed by physicians according to the WHO rules and the ICD 10 coding standards [6, 7]. Data were analyzed using EPI INFO 7 software. ANOVA statistical test was used in our study. Statistical test was only significant if the p value was less than 5% (p≤0.05).

## Results

A total of 970 patients were admitted during our study period. 128 deaths (13.2%) were notified. 54.7% of death (n=70) were male; sex ratio was 1.2. 72.7% of patients who died were residing in Ouagadougou (n=93) and 27.3% were residing outside of Ouagadougou (n=35).

**Age:** Mean age for our study population was 52.8 ±18.3 years with ages ranging from 15 to 98 years. Mean age for patients who died was 56.1 ±19.1 years, with ages ranging from 17 to 87 years while mean age for surviving patient was 52.3 ± 18.1 with ages ranging from 15 to 98 (p= 0.03). Mean age for male patients who died was 58.9 ± 18.7 with ages ranging from 18 to 87 years while the one for female patients was 52.6 ± 19.2 with ages ranging from 17 to 85 (p=0,06).

**Cardiovascular risk factors:** High Blood pressure was noticed in 46.1% (n=59) of cases. Table 1 summarizes the distribution of patients according to cardiovascular risk factors.

**Table 1:** Distribution of patients according to cardiovascular risk factors

	Frequency	Percentage
Hypertension	56	45.1
Alcohol	35	27.3
Smoking	21	16.4
Diabetes	11	08.6
Obesity	03	02.3

**Past Medical History:** Dilated cardiomyopathies were noticed in 27.4% (n=35) of cases. Table 2 summarizes the distribution of patients according to past medical history.

**Table 2:** Distribution of patients according to past medical history

	Frequency	Percentage
Dilated Myocardiopathy	35	27.4
Rheumatic Heart Diseases	19	14.8
Rhythm disorders	12	09.4
Stroke	10	07.8
Ischemic Heart Diseases	09	07
Chronic Obstructive Pulmonary Diseases (COPD)	06	04.7
HIV	05	03.9
Myocardial Infarction	03	02.3

**Time frames:** Mean duration of hospitalization was 10.6 ± 11.6 day with extremes ranging from 01 to 61 days. Mean cardiology inpatient stay was of 26.1 ± 34.6 days with extremes ranging from 01 to 180 days. This mean cardiology stay was of 26.4 ± 34.6 days among patients residing in Ouagadougou with extremes ranging from 01 to 180 days while being of 25.4 ± 34.9 days for those residing outside of Ouagadougou with extremes ranging from 01 to 120 days (p=0.88). The time frame between onset of symptoms and visit to a medical center was of 11.6 ± 21.0 days with extremes ranging from 01 to 120 days. This interval was of 11.7 ± 22.5 days for those living in Ouagadougou with extremes ranging from 01 to 120. This time frame was of 11.4 ± 17.7 days for patients residing outside of Ouagadougou with extremes ranging from 01 to 60 days (p= 0.96).

**Underlying cause of death:** High blood pressure and its complications accounted for 46.1% (n=59) of initial causes of death among hospitalized patients. Table 3 show the distribution of patients according to the initial cause of death. High blood pressure complications included hypertensive cardiomyopathies in 52.5% of cases (N=30). Table 4 summarizes the distribution of complications due to hypertension.

**Table 3:** Distribution of patients according to initial causes of death

	Frequency	Percentage
Hypertensive Heart Disease	34	26.6
Stroke	20	15.6
Ischemic Heart Diseases	17	13.3
Rheumatic Heart Diseases	14	10.9
Infectious Endocarditis	12	09.4
Peri-partum Cardiomyopathy	08	06.3
Pulmonary Embolism	06	04.7
Pericarditis	05	03.9
Sepsis	04	03.1
Cancer	04	03.1
Chronic Pulmonary Vascular Diseases	04	03.1
Total	128	100

**Table 4:** Distribution of hypertensive patients according to hypertension complication

	Frequency	Percentage
Hypertensive Heart Diseases	31	52.5
Stroke	14	23.7
Ischemic Heart Diseases	09	15.3
Rhythm Disorders	03	05.1
Acute Pulmonary Edema	02	03.4
Total	59	100

**Immediate causes of death:** Cardiogenic shock was the immediate cause of death in 55.5% of case. Table 5 summarizes the distribution of patients according to the immediate cause of death.

**Table 5:** Distribution of patients according to immediate cause of death

	Frequency	Percentage
Cardiogenic Shock	71	55.5
Septic Shock	13	10.2
Cerebral Engagement	12	09.4
Ventricular Tachycardia	06	04.7
Pleuro-pneumonia	05	03.9
Hypoglycemia	04	03.1
Complication due to Vitamine K Antagonist	04	03.1
Dyskalemia	04	03.1
Acute Kidney Failure	03	02.3
Hemopericarditis	02	01.6
Liver Failure	02	01.6
Digitalis toxicity	01	00.8
Complete AV block	01	00.8
Total	128	100

**Types of death:** Death was subsequent to treatment in 9.4% of cases (N=12) and to medical error in one case. Death was associated with pregnancy in 8.6% of cases (N=11). No autopsy was performed.

**Certification of Death:** Death was not notified by a physician and notification form was not signed in 18% of cases (N=23). No data could indicate whether an administrative death certificate was ever issued for death cases. None of the medically certified death was made in conformity with WHO recommendations. The summary of inpatient care was the only relevant item found in patients’ medical records. No autopsy was performed.

## Discussion

### Weaknesses

We conducted a retrospective study on patients’ records which did not allow us to gather all necessary data for the study. Determination of the initial cause of death was made manually due to lack of software coding for death causes. This could hinder proper comparison with international studies that use computerized coding.

### Mortality rate

In-hospital mortality due to cardiovascular diseases was 13.2%. This mortality rate is always higher in developing countries compared to developed countries. This disparity is even more pronounced within low income populations [8]. Despite this important lethality due to cardiovascular diseases, we see a delay in starting interventions that would address the problem. In fact, only 39% of African countries compared to 94% in Europe had a program aiming at addressing cardiovascular diseases in the year 2004 [9]. These existing programs however do have difficulties initiating significant health education campaigns due to insufficient resources on top of little involvement of health authorities [9].

### Causes of death

Hypertension was the leading cause of death (46.1%) in our study as well as in other studies in Burkina Faso including one in 1989 (46.2%) [10] and one in 1998 (56.6%) [11]. This observation is due to the fact that patients are often seen at the stage of complications [12, 13], which increases lethality due to Hypertension. Prevalence of in-hospital ischemic heart diseases has increase throughout the years, 1.8% in 1989 [10] in Ouagadougou, 6.1% in 1998 in Bobo-Dioulasso [11], and 13.3% in our study. Coronary heart diseases are difficult to manage in our context, due to delays in diagnosis which precludes early reperfusion using thrombolysis [14], and due to the lack of prevention programs aiming at reducing cardiovascular risk factors. In hospital mortality due to ST Elevation Myocardial Infarction (STEMI) in record studies was of 7% in GRACE and of 9.3% in Euro-Heart Survey [15]. Mortality due to coronary heart diseases (CHD) decreased by 50% from the year 1975 to 2000. This trend is due to both efficient treatments and reduction of cardiovascular risk factors. However the increase in prevalence of diabetes and obesity during the same period slashed the benefit of reduced cardiovascular risk factors on mortality due to CHD by 20% [16]. Generally, mortality due to cardiovascular diseases can be reduced through primary prevention means such as population-based health education programs and early screening of these diseases. However, these interventions require gathering reliable data on mortality and determining the causes of death in an efficient way.

### Death certification

Death was not notified in patients’ records in 18% of cases and death certification was not made in our study. Death notification forms were not similar to the WHO ones. Moreover, administrative death certification was not required prior to burial. Administrative death certification act is only done for inheritance reasons. Data relative to death causes are not used in the process of elaborating action plans for the cardiology department. Statistics on mortality are not reliable in Burkina Faso and in most West African hospitals in which death certification is difficult to perform [4]. South Africa, Egypt, Maurice Islands and Seychelles are the only African countries that keep records of almost all deaths, while mentioning the causes of death. These data are not available in other African countries except for localized areas or portions of their population.

## Conclusion

Mortality due to cardiovascular diseases can be reduced through population based preventive health interventions. However, data on the causes of death which are often used to guide and assess these interventions as well as researches in public health are not reliable in our country. Implementing a system that will record in-hospital deaths and certify death causes will allow generation of reliable statistics on death, measure mortality rate, and focus on specific public health interventions.
